# An investigation of a hemophilia A female with heterozygous intron 22 inversion and skewed X chromosome inactivation

**DOI:** 10.3389/fgene.2024.1500167

**Published:** 2025-01-06

**Authors:** Xiaoyan Tan, Yi Yang, Xia Wu, Jing Zhu, Teng Wang, Huihui Jiang, Shu Chen, Shifeng Lou

**Affiliations:** ^1^ Department of Hematology, The Second Affiliated Hospital, Chongqing Medical University, Chongqing, China; ^2^ Department of Hematology, Three Gorges Hospital, Chongqing University, Chongqing, China

**Keywords:** hemophilia a, females, carriers, x-chromosome inactivation, gene mutations

## Abstract

**Objectives:**

Hemophilia A (HA) is an X-linked recessive inherited bleeding disorder that typically affects men. Women are usually asymptomatic carriers, and rarely presenting with severe or moderately severe phenotype. This study aims to describe a case of a 17-year-old girl with moderate HA, investigating the mechanisms of her condition and the genetic basis within her family.

**Methods:**

We conducted coagulation tests and bleeding assessments to evaluate her bleeding phenotype. Molecular genetic examinations, karyotype analysis, X-chromosome inactivation testing, and targeted bioinformatic analysis were used to identify potential genetic etiologies.

**Results:**

The proband exhibited a severe bleeding phenotype and was found to be a heterozygous carrier of an intron 22 inversion (Inv22) with a normal chromosomal karyotype. No other hemostatic defects were identified through whole exome sequencing. The proband’s mother and monozygotic twin sister are also Inv22 carriers, yet remain asymptomatic with normal FVIII activity. X-chromosome inactivation experiments revealed unbalanced inactivation in the proband, leading to the silencing of the healthy X copy. Notably, several novel X-linked gene mutations (SHROOM2, RPGR, VCX3B, GAGE, GCNA, ZNF280C, CT45A, and XK) were identified in the proband compared to her monozygotic twin sister, though their impact on X-chromosome inactivation remains unclear.

**Conclusion:**

Our findings suggest that the proband’s bleeding phenotype results from unbalanced X-chromosome inactivation. This research marks the first analysis of X chromosome-related gene mutations among monozygotic twins who are carriers of hemophilia A, laying the groundwork for further investigations into the disorder’s pathogenesis in women and highlighting the complexities in genetic counseling.

## 1 Introduction

Hemophilia A (HA) is an X-linked recessive inherited bleeding disorder caused by an abnormal quality or quantity of factor VIII (FVIII). Clinical manifestations include spontaneous bleeding or bleeding after minor trauma in joints, muscles, internal organs, and deep tissues. Repeated joint bleeding can gradually impair joint mobility and lead to disability (Mannucci, 2008; Quintana Paris, 2023). HA primarily affects males, while females are typically asymptomatic carriers, although they may also experience symptoms and rarely exhibit severe or moderately severe clinical phenotypes (Mingot Castellano, 2020; Cygan and Kouides, 2021; Dardik et al., 2023; Hermans et al., 2023). Recently, increasing evidence suggests an increased bleeding tendency in female hemophilia carriers (HCs) (Young et al., 2017; Chaudhury et al., 2020; Li et al., 2020), which warrants further attention and research. To improve diagnosis and management and establish uniform terminologies for clinical research, the International Society on Thrombosis and Haemostasis (ISTH) has developed a new nomenclature that takes into account personal bleeding history and baseline plasma FVIII levels in female patients. This nomenclature distinguishes five clinically relevant HC categories: women/girls with mild, moderate, or severe hemophilia (consistent with the diagnostic criteria for HA in males), symptomatic and asymptomatic HC (van Galen et al., 2021). Currently, there is limited information available in female patients with severe and moderate hemophilia.

The molecular pathogenesis of male HA is well established as a typical FVIII monogenic disease. In approximately 40% of male HA patients, the disorder is attributed to inversions occurring in the F8 gene. The remaining cases are caused by various mutations in F8, including small and large deletions, insertions, as well as non-sense and missense mutations (Borràs et al., 2022). However, the genetic defects underlying female HA exhibit wide variability and few larger reports on its genetic etiology are available. Currently, skewed X-chromosome inactivation (XCI) combined with F8 variants on the active allele is believed to be the most common cause of female HA. Other molecular mechanisms include the involvement of a second mutation in F8 gene (homozygous or compound heterozygous), the presence of a second hemostatic defect due to mutations in genes other than F8 gene (e.g., von Willebrand factor, VWF), abnormalities of the X-chromosome in structure and number (e.g., Turner syndrome), and androgen insensitivity syndrome (Janczar et al., 2020; Miller and Bean, 2020; Shen et al., 2022). Current understandings of hemophilia genetics in female patients are mainly based on published case reports. Therefore, it is necessary to evaluate the clinical characteristics and clarify the pathogenesis for each female HA patient.

Here, we describe a case of a girl with moderate HA, exhibiting a severe bleeding phenotype. We analyzed the laboratory characteristics of the proband and her family members. Additionally, we conducted research on the pathogenesis of the proband through genetic testing for F8, chromosomal karyotype analysis, whole exome sequencing (WES), XCI assay, and X-chromosome targeted bioinformatic analysis.

## 2 Materials and methods

### 2.1 Subjects

Patients and their families were studied after obtaining their written informed consent. The study was conducted in accordance with the Declaration of Helsinki and approved by the Ethics Committee of the Second Affiliated Hospital of Chongqing Medical University.

### 2.2 Haemostatic tests

Basic hemostatic analyses were conducted using the Stago fully automated coagulation analyzer and its corresponding reagents, including prothrombin time (PT), activated partial thromboplastin time (APTT), factor VIII activity (FVIII:C), and plasma von Willebrand factor antigen (vWF:Ag).

### 2.3 Karyotype analysis

Peripheral blood samples were collected from the proband using heparin anticoagulant. The samples were cultured according to standard cytogenetic protocols (Bates, 2011). Giemsa-banding staining was used to analyze the cultured cells, and then the karyotype was analyzed using a chromosome karyotyping software system (Zeiss).

### 2.4 Genetic analysis

The genomic DNA was extracted from peripheral blood using QIAGEN’s QIAamp DNA Blood Mini Kit following the provided instructions. Long-distance PCR ((LD-PCR), gene sequencing, and Multiplex Ligation-dependent Probe Amplification (MLPA) technology was applied to screen for the *F8* mutations. Among these, LD-PCR technology is used to detect the intron 1 and intron 22 inversion mutations of the *F8*; gene sequencing involves direct sequencing of the coding regions of the F8 gene’s exons and comparing them with reference sequences (NM_000132.3 and NG_011403.1) to identify potential micro-mutations; MLPA detection is used to examine each exon of the F8 gene in the sample, using normal human DNA as a reference to determine whether there are deletions or duplications in the gene.The intron 22 inversion (Inv22) was assessed by LD-PCR as per the protocol described in the kit’s instructions (AmpliTaq Gold^®^ 360 Master Mix). The polymorphism of 17 autosomal short tandem repeats (STRs) (THO1, D21S11, D2S1338, Penta E, D5S818, D13S317, D7S820, D16S539, CSF1PO, vWA, D8S1179, TPOX, FGA, D6S1043, D12S391, D10S1248, Penta D) and a sex marker (amelogenin) was analyzed in the DNA samples of the proband and her twin sister. The WES process was carried out by Shanghai Diagnostics Biotechnology Co., Ltd. The sequencing library was constructed using the VAHTS Universal DNA Library Prep Kit for Illumina V3 (NuGEN) and captured using Twist Comprehensive Exome (twist). High-throughput sequencing was performed on the library using the NovaSeq 6,000 (Illumina) sequencer. X chromosome-focused bioinformatics analysis was done based on WES.

### 2.5 X chromosome inactivation

Currently, methylation-based methods are the most widely used for quantitatively defining human XCI status. In this study, we initially utilized the methylation-sensitive HUMARA (human androgen receptor) assay, following previously described modifications (Juchniewicz et al., 2018), to determine XCI patterns across all DNA samples. However, due to the uninformative nature of the androgen receptor locus in this case, we subsequently assessed XCI status at the ZNF261 locus using primers designed by Beever et al. (2003). This approach employed the methylation-sensitive restriction endonuclease HhaI (Thermo Scientific™, ER1851) and a pair of ZNF261-specific fluorescently labeled primers (forward primer: 5′-ATG​CTA​AGG​ACC​ATC​CAG​GA-3’; reverse primer: 5′-GGA​GTT​TTC​CTC​CCT​CAC​CA-3′). DNA was extracted from peripheral blood samples of the proband and family members, and DNA concentration and purity were measured. Each sample (500 ng DNA) was digested with 20 U of HhaI enzyme in a 20 μL reaction buffer at 37°C for 16 h, followed by heat inactivation at 65°C for 20 min. Complete digestion was confirmed by PCR and agarose gel electrophoresis. A 20 μL PCR system was then used to amplify both digested and undigested DNA, and the resulting amplification products were verified using 2% agarose gel electrophoresis. Capillary electrophoresis was performed on a genetic analyzer (Superyears, Classic116, Nanjing, China) to obtain fluorescence values.

To ensure the reliability and accuracy of the results, we strictly adhered to standard operating procedures (SOP) throughout the experiment. To minimize the impact of stutter peaks—a common issue with dinucleotide repeats—we optimized PCR conditions, employed high-resolution capillary electrophoresis, used GeneMapper software for precise data analysis, and conducted repeated experiments. The XCI ratio was calculated based on fluorescence intensities before and after digestion: (a) the paternal X chromosome inactivation ratio was determined as the signal intensity of the paternal X chromosome after digestion divided by its intensity before digestion; (b) the maternal X chromosome inactivation ratio was calculated similarly. XCI patterns were classified into random (50:50 to 70:30), moderately skewed (70:30 to 90:10), or highly skewed (90:10 or above) categories, consistent with criteria established in previous studies (Xiao et al., 2023; Bagislar et al., 2006; Yoon et al., 2008). These rigorous methodologies allowed for accurate classification of XCI patterns in our study.

## 3 Results

### 3.1 Case presentation

The proband is a 17-year-old female with a history of hemorrhage including epistaxis, gingival bleeding, skin bruising, frequent bruising after minor trauma, and menorrhagia. At the age of 13, she experienced a ruptured left ovarian corpus luteum with bleeding and underwent surgical treatment, which was followed by postoperative bleeding. Coagulation assays revealed significantly prolonged APTT, normal PT, factor VIII activity of 1.4%, and normal vWF:Ag levels. Due to having a younger brother with severe HA, she was diagnosed with moderate HA and received replacement therapy with plasma and FVIII concentrates. At the age of 14, she had one episode of gastrointestinal bleeding, requiring FVIII concentrates replacement therapy. Her bleeding score, calculated with the Bleeding Assessment Tool (BAT) (Rodeghiero et al., 2010), was 12, as compared to 0–5 in normal females.

The proband’s maternal grandfather, grandmother, father, mother, and twin sister reported no hemorrhagic symptoms. The proband’s 10-year-old brother is a severe HA patient and requires long-term replacement therapy with FVIII concentrates. The proband’s mother has a younger brother who had recurrent bleeding, joint pain, and joint deformities, but was not definitively diagnosed. He passed away at the age of 6. After obtaining informed consent, we conducted this study on the proband, her parents, twin sister, and brother, while other relatives declined to participate in this study.

### 3.2 Subject characteristics

The coagulation test results of the proband and her family members are presented in Table 1. The proband and her brother had significantly decreased FVIII activity, at 1.1% and 0.4% respectively, while their father, mother, and twin sister had normal FVIII activity. APTT test results were consistent with the FVIII activity test results, with the brother showing significantly prolonged APTT, followed by the proband, while the father, mother, and sister had normal results. The level of vWF:Ag of the proband and her family members was within the normal range. Both the proband and her brother tested negative for FVIII inhibitor. Additionally, targeted joint ultrasound examinations were performed on the proband and her brother, including the elbows, knees, and ankles. The results showed that the proband’s joint ultrasound examination was normal, but her brother had varying degrees of hemophilic arthropathy changes in both knees and ankles (data not shown).

**TABLE 1:** Laboratory Findings of the proband and her family.

Family member	Father	Mother	Proband	Sister	Brother	Reference values
Age (years)	37	37	17	17	10	
FⅧ activity (%)	121	68.9	1.1	62	0.4	60–150
FIX activity (%)	90	91.9	82	82	85	60–150
APTT (sec)	42.5	41.2	85.8	45.7	93.8	28–44
vWF:Ag (%)	109	114	105	93	87	50–160
Inhibitor	-	-	negative	-	negative	negative
RBC(10^12^/L)	4.55	4.4	4.94	4.74	4.33	Female: 3.8–5.1 Male: 4.3–5.8
WBC(10^9^/L)	5.92	4.6	6.38	4.11	7.23	3.5–9.5
PLT (10^9^/L)	230	208	142	124	160	100–300

APTT, activated partial thromboplastin time; FVIII, activity: factor VIII, activity; vWF:Ag, plasma von Willebrand factor antigen; RBC, red blood cell; WBC, white blood cell; PLT, platelet; “-” represents “Not tested”.

### 3.3 Gene and chromosome analysis

Genetic screening for HA revealed a heterozygous Inv22 of the *F8* in the proband. To determine if there are other chromosomal or genetic abnormalities exacerbating the deficiency of FVIII, we performed chromosomal karyotype analysis and WES on the proband. The patient’s chromosomal karyotype analysis was normal, and WES revealed no other mutations associated with bleeding disorders (Table 2).

**TABLE 2:** Exome sequencing findings in proband and her monozygotic twin sister.

Proband/Twin sister	Gene symbol	Chromosomal location	Mutation location	Variant change	Zygosity status	ACMG classification	Associated diseases
Proband	LYST	chr1:235,971,946	exon5	c.A2172G: p. (Ile724Met)	Heterozygous	Uncertain significance	Chediak-Higashi syndrome
Proband	NBEAL2	chr3:47,036,532	exon13	c.G1307T: p. (Gly436Val)	Heterozygous	Uncertain significance	Gray Platelet Syndrome
Twin Sister	DYSF	chr2: 71,742,844	exon7	c.755C>T p. (Thr252Met)	Heterozygous	Likely Pathogenic	Muscular dystrophy
Twin Sister	IFT74	chr9: 27,036,462	exon14	c.1069G>T p. (Gly357*)	Heterozygous	Uncertain significance	Bardet-Biedl syndrome
Twin Sister	PRPH	chr12: 49,690,678	exon4	c.709C>T p. (Arg237*)	Heterozygous	Likely Pathogenic	Amyotrophic Lateral Sclerosis
Twin Sister	RRM2 B	chr8: 103,250,919	exon1	c.132G>A p. (Trp44*)	Heterozygous	Likely Pathogenic	Mitochondrial DNA depletion syndrome
Twin Sister	SARS2	chr19: 39,412,301	exon4	c.399dupC p. (Lys134Glnfs *27)	Heterozygous	Likely Pathogenic	Hyperuricemia - Pulmonary Hypertension - Renal Failure - Alkalosis Syndrome

ACMG, american college of medical genetics and genomics.

**FIGURE 1:**
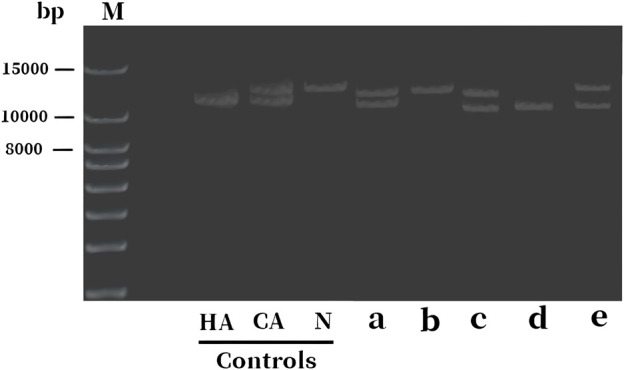
Electrophoresis diagram of *F8* gene Inv22 detection in the proband and their family members. The bands 11 and 12 kb long identify the Inv22 and wild-type alleles, respectively. The lanes a to e represent samples from the proband, father, mother, brother, and sister, respectively. The results indicate that the proband, mother, and the sister are HA carriers, while the brother is a HA patient, and the father is normal. HA: hemophilia A; CA, carrier; N, normal subject; Inv22, intron 22 inversion; M: DNA molecular weight marker.

**FIGURE 2:**
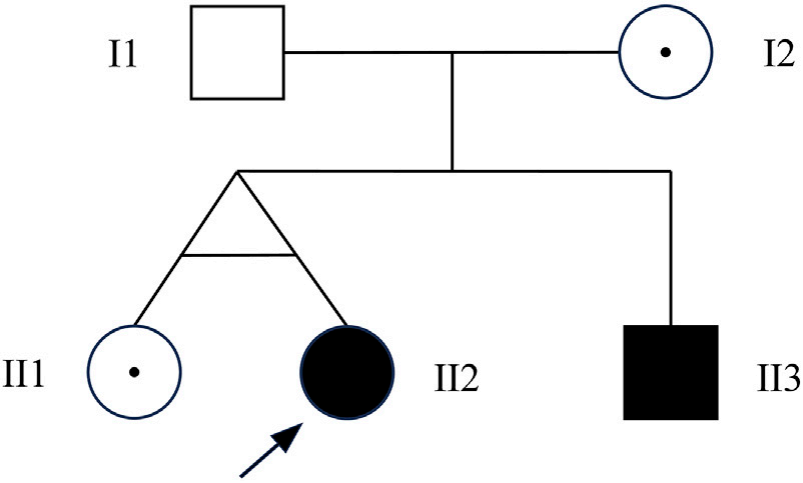
Pedigree chart. I1: normal male; I2: female Inv22 carrier; II1: female Inv22 carrier; II2: the proband, female Inv22 carrier, but presented as HA phenotype; III3: male with Inv22, a HA patient. Inv22: intron 22 inversion; HA, hemophilia A.

To confirm the inheritance mode of the Inv22 in the family, we further validated it in the proband’s parents, sister, and brother. Specific primers were used to amplify the gene fragment flanking the Inv22, and the analysis was performed using agarose gel electrophoresis. The results showed that two bands were obtained from the samples of the proband, proband’s mother, and sister (one 12 kb long identifying the normal allele, the other 11 kb long identifying the Inv22 mutated allele), which is characteristic of Inv22 carrier. The father’s sample showed one band at the 12 kb position, indicating a normal state. The brother showed an amplification band only at the 11 kb position, indicating a HA patient (Figure 1). The results of the STRs polymorphism analysis indicated that the proband and her twin sister are monozygotic twins (Supplementary Table S1). The pedigree diagram is shown in Figure 2.

### 3.4 X chromosome inactivation analysis

**FIGURE 3:**
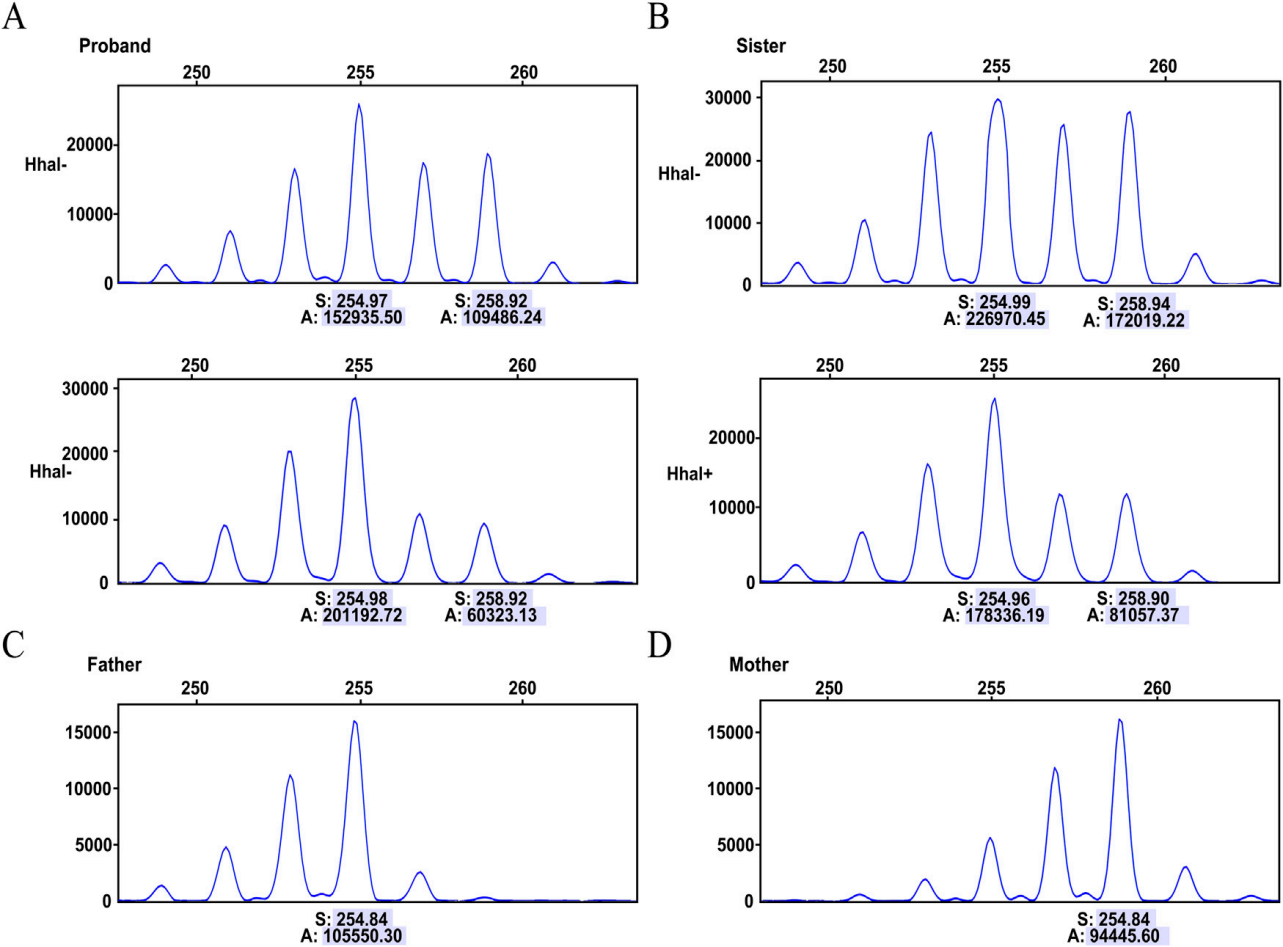
Capillary electrophoresis of the X chromosome inactivation analysis. Digesting with the HhaI restriction enzyme, which selectively cuts active (non-methylated) DNA. **(A)** Analysis of the ZNF261 polymorphism in the proband, displaying results before digestion (HhaI-) and after digestion (HhaI+). **(B)** Analysis of the ZNF261 polymorphism in the monozygotic twin sister, displaying results before digestion (HhaI-) and after digestion (HhaI+). **(C)** The 254 bp allele identifies the normal X chromosome inherited from father to daughter. **(D)** The 258 bp ZNF261 allele identifies the Inv22 X chromosome inherited from mother to daughter. The numbers below each allele represent peak areas **(A)** and base pair sizes (S). The relative percentage of active and inactivated X chromosome was calculated from the ratio of the peak areas of the different ZNF261 alleles before and after digestion.

The proband is an Inv22 carrier, and her FVIII levels decreased enough to classify her as a case of moderate HA. Therefore, she was tested for non-random XCI, a condition that may lead to this phenotypic expression of HA in female carriers. Initially, we analyzed the polymorphic CAG locus in the HUMARA gene in the genomic DNA of the proband and her sister before and after digestion with the methylation-sensitive restriction endonuclease HhaI. The results show that the amplifications of the HUMARA alleles from both the father and mother of the proband have the same fragment length of 272 bp, making it impossible to determine the XCI status. The same result was found for her sister (data not shown).Therefore, we changed our approach and analyzed the polymorphic locus of the ZNF261 gene. The ZNF261 PCR fragment, 258 bp long, was inherited from the mother and identified the X chromosome carrying the Inv22 mutation. The 258 bp allele in the proband was mostly digested, while her second ZNF261 allele (254 bp in length, inherited from the father, identifying the X chromosome carrying the normal F8 allele) showed almost no change after HhaI digestion (Figure 3). HhaI restriction enzymes can selectively cut active (non-methylated) DNA, so the above results suggest that the proband’s Inv22 X chromosome was transcriptionally very active, while her normal X copy was silenced (Inv22: wild-type ratio 71:29, moderately skewed). A more balanced X inactivation ratio was observed in the proband’s sister, with an Inv22: wild-type ratio of 63:37 (Figure 3).

### 3.5 X-chromosome targeted bioinformatic analysis

To investigate whether other X chromosome-related gene mutations led to XCI skewing in the proband, we conducted targeted bioinformatics analysis of the WES results focusing on the X chromosome. The results were then compared between the proband and her monozygotic twin sister. The proband identified several novel X chromosome-related mutations, including SHROOM2, RPGR, VCX3B, GAGE, GCNA, ZNF280C, CT45A, and XK. Comprehensive information about these mutated genes is available in Table 3. These genes are associated with the development of tissues such as the nervous system, retina, sperm, and blood, as well as tumor occurrence (according to NCBI and ClinVar). Currently, none of these mutations have been reported to result in skewed XCI.

**TABLE 3:** Novel X chromosome-related mutations identified in the proband compared to her monozygotic twin sister.

Gene symbol	Chromosomal location	Mutation location	Variant locus	Mutation frequency
SHROOM2	chrX:9,859,132	exon3	c.G433A:p. (Gly145Ser)	52.86%
RPGR	chrX:38,145,130	exon15	c.A3122G:p. (Glu1041Gly)	30.00%
VCX3B	chrX:8,434,251	exon3	c.G568A:p. (Val190Met)	22.09%
GAGE	chrX:49,221,278	exon4	c.C223G:p. (His75Asp)	13.13%
GCNA	chrX:70,823,980	exon8	c.T853C:p. (Ser285Pro)	9.68%
ZNF280C	chrX:129,349,888	exon14	c.C1715A:p. (Ala572Glu)	6.20%
CT45A	chrX:134,889,973	exon4	c.G441T:p. (Met147Ile)	4.35%
XK	chrX: 37,587,031	exon3	c.T651G:p. (Phe217Leu)	3.95%

## 4 Discussion

This is the case of our proband, an Inv22 carrier with skewed XCI, whose FVIII levels decreased enough to classify her as a case of moderate HA, and therefore representing one of the extremely rare cases of the disease that have been described in females. The proband exhibited significant bleeding symptoms and had a family history of HA, yet the diagnosis of HA did not occur until a severe bleeding event at the age of 13. This delayed diagnosis indicates the need for increased attention to individuals in similar situations. Referring to the study by Daidone et al. (2018), we utilized the BAT questionnaire, a tool currently employed in von Willebrand’s disease, to assess the proband’s bleeding tendency. Her bleeding score was found to be significantly higher than normal. Since HA carriers are typically asymptomatic or have mild symptoms, evaluating the bleeding score could aid in identifying these patients in specific populations, particularly in females with a family history. This approach may also help in better defining their bleeding risk and enable more appropriate management. Therefore, it would be valuable to incorporate the BAT questionnaire into clinical practice for this purpose.

The proband has a younger brother with severe HA and a healthy twin sister, making it essential to conduct a family pedigree study. Unfortunately, apart from the proband’s parents, sister, and brother, all other relatives have refused to participate in the investigation. The family investigation revealed that the proband’s mother transmitted Inv22 mutation to her three children. More interestingly, it was confirmed that the proband and her sister are monozygotic twins. However, even though they carry the same mutation, the phenotypes are different. Thus, we speculate that the proband may have acquired chromosomal abnormalities or skewed XCI during development. These two conditions have been previously observed in females with HA and may explain the manifestation in heterozygous mutation carriers (Knobe et al., 2008; Berendt et al., 2020; Janczar et al., 2020). Karyotype analysis revealed the proband to have a normal 46, XX karyotype, eliminating the diagnosis of Turner Syndrome or other large chromosomal abnormalities. Subsequently, XCI analysis explained the different phenotypes between the monozygotic twins. Currently, there are only a few case reports on the occurrence of HA in female monozygotic twins (Valleix et al., 2002; Bennett et al., 2008). One case report describes a pair of monozygotic twins, with only one showing the phenotype of HA, while the other is an asymptomatic carrier. Another report describes a pair of monozygotic twins, both presenting with HA. The reported mechanisms involve unbalanced XCI leading to the overexpression of mutant alleles. However, the potential mechanisms behind the differences in XCI between monozygotic twins remain unclear.

In the normal female population, about 10% of females exhibit non-random XCI in peripheral blood cells. Non-random XCI typically does not have an impact on females. However, in certain cases, skewed XCI can result in X-linked disease in females due to a prevalent silencing of the healthy X copy (Khan and Theunissen, 2023; Schwämmle and Schulz, 2023). Currently, the relationship between FVIII activity and the severity of XCI skewing in female HA carriers has not been well elucidated. Additionally, there is a variety of criteria in the research field for defining XCI deviation. Unlike the previously published studies (Knobe et al., 2008; Daidone et al., 2018), female HA patients showed strongly unbalanced XCI. However, in our case, the maternal XCI ratio of Inv22 was 29%, indicating a moderate inactivation shift, yet the FVIII activity was very low, at only 1.1%. We need to pay attention to the limitations of the XCI marker. The dinucleotide repeat sequence of ZNF261 has high polymorphism as an XCI analysis marker; however, it may lead to stutter peaks, which can affect the results of XCI. In this study, to ensure the reliability of the results, we have optimized the experimental conditions, employed high-resolution capillary electrophoresis to better distinguish stutter peaks, and conducted repeated experiments. Therefore, we still believe that ZNF261 can provide sufficient accuracy for determining the XCI pattern in our research. Given the limitations of HUMARA in our study, selecting other suitable XCI markers for more comprehensive validation will be more helpful in ensuring the reliability and accuracy of the data. FVIII is mainly produced by liver cells; however, studies have shown that non-random XCI does not differ significantly in different tissues and is mainly influenced by age (Bittel et al., 2008). Thus, we cannot completely rule out the possibility that other factors may be contributing to the decreased FVIII activity in the proband, such as deep intronic mutations. Additional tests like RNA sequencing and deep intronic sequencing will aid in clarifying these matters.

In addition, there are many reasons for non-random XCI, which can be classified as primary or secondary. Primary non-random XCI occurs at the initial stage of inactivation. Secondary non-random X chromosome inactivation is often related to cell selection, where the presence of certain specific gene mutations on the X chromosome can lead to cells gaining a proliferative advantage or disadvantage (Dardik et al., 2021; Dardik et al., 2023). In our study, all three females were Inv22 carriers, but only the proband experienced unbalanced XCI. Analysis of the WES results of the proband and her monozygotic twin sister revealed the presence of some novel gene mutations, with the SHROOM2 mutation frequency exceeding 50%. However, currently, there are no reports linking any of these gene mutations to XCI skewing. The limitation of this study is the lack of functional validation for these mutations. Therefore, the impact of these different gene mutations between monozygotic twins on XCI and FVIII activity remains unclear. However, we believe that our findings provide important clues and potential directions for further functional validation studies. We believe that future work will further clarify the relationship between these mutations and the pathogenesis of HA, providing deeper insights into the role of XCI in female patients with HA.

## 5 Conclusion

We report a rare case of a girl with moderate HA carrying a heterozygous Inv22 mutation. Pedigree analysis revealed that the proband’s mother and her monozygotic twin sister are both Inv22 carriers, but only the proband exhibited severe bleeding symptoms. Chromosome inactivation analysis indicated that the proband’s clinical phenotype was attributed to an unbalanced XCI pattern, resulting in the allele carrying the Inv22 being transcriptionally more active than the normal allele. Furthermore, we identified several novel X-linked gene mutations, including SHROOM2, RPGR, VCX3B, GAGE, GCNA, ZNF280C, CT45A, and XK, in the proband compared to her monozygotic twin sister. However, it remains uncertain whether these mutations affect XCI in the proband. We conducted the first analysis of the differences in X chromosome-related gene mutations among monozygotic twins who are carriers of HA, laying the foundation for further research into the pathogenesis of HA in women. Our study also emphasizes that the complexity of the pathogenesis of HA in females poses challenges for subsequent genetic counseling.

## Data Availability

The data presented in the study are deposited in the Genome Sequence Archive (Genomics, Proteomics and Bioinformatics 2021) in the National Genomics Data Center (Nucleic Acids Res 2024), China National Center for Bioinformation/Beijing Institute of Genomics, Chinese Academy of Sciences, accession number GSA-Human: HRA009718, and are publicly accessible at https://ngdc.cncb.ac.cn/gsa-human.

## References

[B1] Bagislar S. Ustuner I. Cengiz B. Soylemez F. Akyerli C. B. Ceylaner S. (2006). Extremely skewed X-chromosome inactivation patterns in women with recurrent spontaneous abortion. Aust. N. Z. J. Obstet. Gynaecol. 46 (5), 384–387. 10.1111/j.1479-828X.2006.00622.x 16953851

[B2] Bates S. E. (2011). Classical cytogenetics: karyotyping techniques. Methods Mol. Biol. 767, 177–190. 10.1007/978-1-61779-201-4_13 21822875

[B3] Beever C. Lai B. P. Y. Baldry S. E. L. Peñaherrera M. S. Jiang R. Robinson W. P. (2003). Methylation of ZNF261 as an assay for determining X chromosome inactivation patterns. Am. J. Med. Genet. Part A 120A (3), 439–441. 10.1002/ajmg.a.20045 12838571

[B4] Bennett C. M. Boye E. Neufeld E. J. (2008). Female monozygotic twins discordant for hemophilia A due to nonrandom X‐chromosome inactivation. Am. J. Hematol. 83 (10), 778–780. 10.1002/ajh.21219 18645989 PMC5715470

[B5] Berendt A. Wójtowicz-Marzec M. Wysokińska B. Kwaśniewska A. (2020). Severe haemophilia a in a preterm girl with turner syndrome - a case report from the prenatal period to early infancy (part I). Italian J. Pediatr. 46 (1), 125. 10.1186/s13052-020-00892-7 PMC7487698 32894158

[B6] Bittel D. C. Theodoro M. F. Kibiryeva N. Fischer W. Talebizadeh Z. Butler M. G. (2008). Comparison of X-chromosome inactivation patterns in multiple tissues from human females. J. Med. Genet. 45 (5), 309–313. 10.1136/jmg.2007.055244 18156436 PMC5489244

[B7] Borràs N. Castillo-González D. Comes N. Martin-Fernandez L. Rivero-Jiménez R. A. Chang-Monteagudo A. (2022). Molecular study of a large cohort of 109 haemophilia patients from Cuba using a gene panel with next generation sequencing-based technology. Haemophilia 28 (1), 125–137. 10.1111/hae.14438 34708896

[B8] Chaudhury A. Sidonio R. Jain N. Tsao E. Tymoszczuk J. Oviedo Ovando M. (2020). Women and girls with haemophilia and bleeding tendencies: outcomes related to menstruation, pregnancy, surgery and other bleeding episodes from a retrospective chart review. Haemophilia 27 (2), 293–304. 10.1111/hae.14232 33368856 PMC8220814

[B9] Cygan P. H. Kouides P. A. (2021). Regulation and importance of factor VIII levels in hemophilia A carriers. Curr. Opin. Hematol. 28 (5), 315–322. 10.1097/moh.0000000000000667 34397591

[B10] Daidone V. Galletta E. Bertomoro A. Casonato A. (2018). The Bleeding Assessment Tool and laboratory data in the characterisation of a female with inherited haemophilia A. Blood Transfus. 16 (1), 114–117. 10.2450/2016.0132-16 27893350 PMC5770322

[B11] Dardik R. Avishai E. Lalezari S. Barg A. A. Levy-Mendelovich S. Budnik I. (2021). Molecular mechanisms of skewed X-chromosome inactivation in female hemophilia patients-lessons from wide genome analyses. Int. J. Mol. Sci. 22 (16), 9074. 10.3390/ijms22169074 34445777 PMC8396640

[B12] Dardik R. Janczar S. Lalezari S. Avishai E. Levy-Mendelovich S. Barg A. A. (2023). Four decades of carrier detection and prenatal diagnosis in hemophilia A: historical overview, state of the art and future directions. Int. J. Mol. Sci. 24 (14), 11846. 10.3390/ijms241411846 37511607 PMC10380558

[B13] Hermans C. Ventriglia G. Obaji S. Beckermann B. M. Lehle M. Catalani O. (2023). Emicizumab use in females with moderate or mild hemophilia A without factor VIII inhibitors who warrant prophylaxis. Res. Pract. Thromb. Haemost. 7 (8), 102239. 10.1016/j.rpth.2023.102239 38193069 PMC10772889

[B14] Janczar S. Babol-Pokora K. Jatczak-Pawlik I. Taha J. Klukowska A. Laguna P. (2020). Six molecular patterns leading to hemophilia A phenotype in 18 females from Poland. Thrombosis Res. 193, 9–14. 10.1016/j.thromres.2020.05.041 32497951

[B15] Juchniewicz P. Kloska A. Tylki-Szymańska A. Jakóbkiewicz-Banecka J. Węgrzyn G. Moskot M. (2018). Female Fabry disease patients and X-chromosome inactivation. Gene 641, 259–264. 10.1016/j.gene.2017.10.064 29079200

[B16] Khan S. A. Theunissen T. W. (2023). Modeling X-chromosome inactivation and reactivation during human development. Curr. Opin. Genet. Dev. 82, 102096. 10.1016/j.gde.2023.102096 37597506 PMC10588740

[B17] Knobe K. E. SjÖRin E. Soller M. J. LiljebjÖRn H. Ljung R. C. R. (2008). Female haemophilia A caused by skewed X inactivation. Haemophilia 14 (4), 846–848. 10.1111/j.1365-2516.2008.01754.x 18540890

[B18] Li S. Fang Y. Li L. Lee A. Poon M. C. Zhao Y. (2020). Bleeding assessment in haemophilia carriers-High rates of bleeding after surgical abortion and intrauterine device placement: a multicentre study in China. Haemophilia 26 (1), 122–128. 10.1111/hae.13889 31742836

[B19] Mannucci P. M. (2008). Back to the future: a recent history of haemophilia treatment. Haemophilia 14 (Suppl. 3), 10–18. 10.1111/j.1365-2516.2008.01708.x 18510516

[B20] Miller C. H. Bean C. J. (2020). Genetic causes of haemophilia in women and girls. Haemophilia 27 (2), e164–e179. 10.1111/hae.14186 33314404 PMC8132474

[B21] Mingot Castellano M. E. (2020). General concepts on hemophilia A and on women carrying the disease. Blood Coagul. Fibrinolysis 31 (1s), S1–s3. 10.1097/mbc.0000000000000984 33351492

[B22] Quintana Paris L. (2023). Foundations of hemophilia and epidemiology. Blood Coagul. Fibrinolysis 34 (S1), S35–s36. 10.1097/mbc.0000000000001222 37254731

[B23] Rodeghiero F. Tosetto A. Abshire T. Arnold D. M. Coller B. James P. (2010). ISTH/SSC bleeding assessment tool: a standardized questionnaire and a proposal for a new bleeding score for inherited bleeding disorders. J. Thromb. Haemost. 8 (9), 2063–2065. 10.1111/j.1538-7836.2010.03975.x 20626619

[B24] Schwämmle T. Schulz E. G. (2023). Regulatory principles and mechanisms governing the onset of random X-chromosome inactivation. Curr. Opin. Genet. Dev. 81, 102063. 10.1016/j.gde.2023.102063 37356341 PMC10465972

[B25] Shen M. C. Chang S. P. Lee D. J. Lin W. H. Chen M. Ma G. C. (2022). Skewed X-chromosome inactivation and parental gonadal mosaicism are implicated in X-linked recessive female hemophilia patients. Diagn. (Basel) 12 (10), 2267. 10.3390/diagnostics12102267 PMC9600608 36291957

[B26] Valleix S. Vinciguerra C. Lavergne J.-M. Leuer M. Delpech M. Negrier C. (2002). Skewed X-chromosome inactivation in monochorionic diamniotic twin sisters results in severe and mild hemophilia A. Blood 100 (8), 3034–3036. 10.1182/blood-2002-01-0277 12351418

[B27] van Galen K. P. M. d'Oiron R. James P. Abdul-Kadir R. Kouides P. A. Kulkarni R. (2021). A new hemophilia carrier nomenclature to define hemophilia in women and girls: communication from the SSC of the ISTH. J. Thromb. Haemost. 19 (8), 1883–1887. 10.1111/jth.15397 34327828 PMC8361713

[B28] Xiao X. Yang J. Li Y. Yang H. Zhu Y. Li L. (2023). Identification of a novel frameshift variant of ARR3 related to X-linked female-limited early-onset high myopia and study on the effect of X chromosome inactivation on the myopia severity. J. Clin. Med. 12 (3), 835. 10.3390/jcm12030835 36769483 PMC9917903

[B29] Yoon S. H. Choi Y. M. Hong M. A. Kang B. M. Kim J. J. Min E. G. (2008). X chromosome inactivation patterns in patients with idiopathic premature ovarian failure. Hum. Reprod. 23 (3), 688–692. 10.1093/humrep/dem415 18182395

[B30] Young J. E. Grabell J. Tuttle A. Bowman M. Hopman W. M. Good D. (2017). Evaluation of the self-administered bleeding assessment tool (Self-BAT) in haemophilia carriers and correlations with quality of life. Haemophilia 23 (6), e536–e538. 10.1111/hae.13354 28949433

